# Recurrence Risk after Radical Colorectal Cancer Surgery—Less Than before, But How High Is It?

**DOI:** 10.3390/cancers12113308

**Published:** 2020-11-09

**Authors:** Erik Osterman, Klara Hammarström, Israa Imam, Emerik Osterlund, Tobias Sjöblom, Bengt Glimelius

**Affiliations:** 1 Department of Immunology, Genetics and Pathology, Uppsala University, 751 85 Uppsala, Sweden; erik.osterman@igp.uu.se (E.O.); klara.hammarstrom@igp.uu.se (K.H.); israa.imam@igp.uu.se (I.I.); emerik.osterlund@gmail.com (E.O.); tobias.sjoblom@igp.uu.se (T.S.); 2 Department of Surgery, Gävle Hospital, 801 87 Gävle, Sweden

**Keywords:** colorectal cancer, colon cancer, rectal cancer, chemotherapy, adjuvant treatment, recurrence risk, systematic overview

## Abstract

**Simple Summary:**

Evidence indicates that recurrence risk after colon cancer today is less than it was when trials performed decades ago showed that adjuvant chemotherapy reduces the risk and prolong disease-free and overall survival. After rectal cancer surgery, local recurrence rates have decreased but it is unclear if systemic recurrences have. After a systematic review of available literature reporting recurrence risks after curative colorectal cancer surgery we report that the risks are lower today than they were in the past and that this risk reduction is not solely ascribed to the use of adjuvant therapy. Adjuvant therapy always means overtreatment of many patients, already cured by the surgery. Fewer recurrences mean that progress in the care of these patients has happened but also that the present guidelines giving recommendations based upon old data must be adjusted. The relative gains from adding chemotherapy are not altered, but the absolute number of patients gaining is less.

**Abstract:**

Adjuvant chemotherapy aims at eradicating tumour cells sometimes present after radical surgery for a colorectal cancer (CRC) and thereby diminish the recurrence rate and prolong time to recurrence (TTR). Remaining tumour cells will lead to recurrent disease that is usually fatal. Adjuvant therapy is administered based upon the estimated recurrence risk, which in turn defines the need for this treatment. This systematic overview aims at describing whether the need has decreased since trials showing that adjuvant chemotherapy provides benefits in colon cancer were performed decades ago. Thanks to other improvements than the administration of adjuvant chemotherapy, such as better staging, improved surgery, the use of radiotherapy and more careful pathology, recurrence risks have decreased. Methodological difficulties including intertrial comparisons decades apart and the present selective use of adjuvant therapy prevent an accurate estimate of the magnitude of the decreased need. Furthermore, most trials do not report recurrence rates or TTR, only disease-free and overall survival (DFS/OS). Fewer colon cancer patients, particularly in stage II but also in stage III, today display a sufficient need for adjuvant treatment considering the burden of treatment, especially when oxaliplatin is added. In rectal cancer, neo-adjuvant treatment will be increasingly used, diminishing the need for adjuvant treatment.

## 1. Introduction

Colorectal cancer (CRC) is the fourth most common cancer worldwide and the number two cause of cancer death [1]. In early stages (stages I–III), constituting 75–80% of newly diagnosed cases, adjuvant chemotherapy is often administered since it may kill sub-clinical disease and, thereby, decrease the risk of recurrence and improve survival. After colon cancer surgery, it is routine therapy in stage III and in stage II with risk factors for recurrence [2,3,4,5,6], whereas it is less established in rectal cancer, particularly if pre-operative radiotherapy/chemoradiotherapy (RT/CRT) has been administered. The randomized rectal cancer trials have not unequivocally shown enough benefit [7,8]. Despite this, some guidelines recommend the same treatment as in colon cancer [9]. Increased use of adjuvant chemotherapy is one reason for the improved overall survival (OS) for CRC patients observed in cancer registries [10]. Other reasons are linked to screening/earlier detection, improved general health allowing preoperative treatments and surgery in more patients and improved pre- and postoperative care [11,12,13,14,15,16,17,18].

Evidence indicates that the recurrence risks after CRC surgery have decreased due to improvements in care other than that provided by adjuvant therapy [19,20]. Better staging with computed tomography (CT) and magnetic resonance imaging (MRI) of the thorax, abdomen and pelvis will detect smaller distant metastases, resulting in fewer recurrences in those operated [14]. Improved surgical techniques, both in rectal cancer [21], and later also in colon cancer [14,22,23], further reduce recurrence risks. Improved examination of the surgical specimen does not per se reduce recurrence risks, but results in fewer recurrences in each pathological stage, i.e., stage migration [24,25]. An international congress in 1990 recommended that at least 12 nodes should be investigated to properly define the TN-stage [26]. With time, the quality of the pathological examinations, including reporting of the number of investigated nodes, has improved [27]. High numbers of investigated nodes correlate with better outcome in both stage II and III [28,29,30].

In an analysis of the Adjuvant Colon Cancer Endpoints (ACCENT) collaborative group database, recurrence risks were several percentage points lower in patients enrolled since 2000 compared with those enrolled earlier and shown in a nomogram based upon trial data, Adjuvant! Online [31]. It was stated that “the last 15 years have produced an overall improvement in patient outcomes due to a number of different factors including optimization of surgery”, and that “many calculators (of recurrence risk) reflect older practice”. Similar results were seen in another ACCENT study [32], noting signs of stage migration in stage II but not in stage III, with improved outcomes in newer-era trials (initiated patient inclusion between 1995–2000 vs 1978–1993). They “called into question historical data related to the benefit of FU-based adjuvant therapy in such (stage II) patients”. In a third ACCENT database analysis [33], patients did better in the trials including patients between 2004–2009 than in those including patients between 1998–2003, but this was probably explained by better possibilities of tolerating oxaliplatin-containing chemotherapy than reflecting decreased recurrence risks as in the two previous analyses. In 2008, The Memorial Sloan Kettering Cancer Center (MSKCC, www.mskcc.org) published a nomogram describing the risk of recurrence and survival in colon cancer stages I–III based upon patients operated between 1990–2000 [34]. Although validated by others [35,36,37], lower recurrence risks were seen in an update in patients operated between 2007–2014 [38]. In a recent secondary analysis of an adjuvant trial exploring the potential benefit of adding bevacizumab to chemotherapy, the AVANT trial, excellent results were seen suggesting that “the definition of high-risk stage II needs to be revisited” [39].

This review aims at describing the present recurrence rates in patients radically operated for a primary non-metastatic CRC, i.e., that they have cancer cells that will develop into metastases provided the patient lives long enough and does not receive adjuvant treatment. This question is important for many patients and their doctors approximately one month after surgery has taken place. In stage II without any risk factors, adjuvant treatment is not given, but one must ask whether the recurrence risk is sufficiently high to merit treatment if one or two risk factors are present, when guidelines recommend therapy [4,9,40]? Do all stage III patients have such a high risk that an oxaliplatin combination is motivated? When is the risk of recurrence in stage III so high that six months of oxaliplatin is motivated? The elderly and patients with co-morbidities may need better knowledge to weigh the increased risks against the benefits involved [41].

Due to the lack of firm data in literature, a second aim of this review is to describe stage-specific recurrence risks in a Swedish CRC population between 2010 and 2018 where validation of whether patients in stage I–III are recurrence-free or not has been carried out.

## 2. Methodological Considerations

### 2.1. Time-To Event Endpoints

In adjuvant trials, the gains have been recorded as improved OS, being the ultimate goal of these interventions, or as disease-free survival (DFS), balancing gains (fewer recurrences) with significant losses (any deaths and secondary malignancies) [42,43]. However, adjuvant chemotherapy cannot improve OS or DFS without reducing the recurrence risk. Thus, recurrence risk (or freedom from recurrence, FFR) or improved time to recurrence (TTR) is probably the most direct measure of the effect of adjuvant chemotherapy. However, this is rarely recorded in trials and has been used as a primary endpoint in only one trial [44]. TTR ought also to be the most informative endpoint in tumour marker studies but is again rarely used. Although neither TTR nor FFR are ideal methods of estimating the risk of remaining sub-clinical tumour cells, they are more adequate than other time-to-event outcomes. However, even if all endpoints including deaths (even toxic deaths) tend to overestimate “this need”, all endpoints not including deaths may underestimate the risk if the death occurs before the event, i.e., the recurrence. This competing risk is most pronounced in elderly patients but not in the average trial patient where median age lies between 62–65 years. However, most recurrences come early or within the first 3–4 years whereas short-term survival even for very old persons who have undergone major CRC surgery is clearly longer [41]. Toxic deaths are fortunately rare and, in most studies, less than one per cent [45,46]. In the estimation of TTR, all deaths except death from the same cancer, here CRC, are censored [42,43], making TTR a better estimate than FFR estimated from the crude number of recurrences after a specific time. Most of the older adjuvant trials published, besides OS, only report crude recurrence rates. This is also the case in several more recent studies of surveillance strategies or surgical techniques (to be described below) Recurrence-free survival (RFS) also includes all deaths, but not secondary other malignancies [42,43]. With time, secondary malignancies included in DFS but not in RFS increasingly contribute to the number of events in an aged CRC population [47]. The relative importance of secondary malignancies will also increase when recurrences become fewer [48]. Although endpoints have been clearly defined, they have not always been used properly, hampering any literature evaluation.

### 2.2. Representativity

Patients included in clinical trials are not representative of the background population [49,50,51]. Population-based registries seldom include recurrence data, and if they do, they are unreliable, underestimating the risks [52,53]. For example, in two US health care databases, only 7% recurrences were registered in a mixed population of CRC patients operated between 1995–2014 [54]. Lack of registration may also reflect difficulties involved with adequate follow-up. In a large prospective cohort of 15,096 colon cancer patients with stage I + II from 346 German hospitals, only 68% of the patients had a satisfactory follow-up [55]. In the study, 5% developed a local relapse and 10% developed distant metastases, numbers that apparently are too low considering that recurrence risks are generally at least twice as high. OS is properly recorded in registries but overestimate the need for adjuvant therapy since many deaths in individuals with a median age of 70 years (62 in the trials) can never be prevented by adjuvant chemotherapy.

### 2.3. Selective Delivery of Adjuvant Therapy

Since adjuvant chemotherapy is today routine therapy for many patients with colon cancer [4,40], the recurrence risks in patient cohorts collected during the past decades being lower than if this had not been administered. This will falsely decrease the need. Furthermore, patients not treated with adjuvant therapy are selected, often to a poorer alternative with poorer OS and DFS than patients selected for treatment [56]. Whether recurrence risks (or TTR) are also biased is not known. The situation is slightly different in rectal cancer where there is less evidence of favourable effects, and adjuvant therapy is not always given [2,57,58]. In rectal cancer, two recent randomized trials have included a postoperatively non-treated group providing up-to-date recurrence rates after preoperative treatment [59,60]. In colon cancer, a surgery alone group has since the 1990s only been included in one Japanese study including stage II patients [48].

Recent trials on surveillance strategies or comparisons of different surgical techniques are a source of information, but if recent, they are performed at hospitals providing adjuvant chemotherapy for sub-groups. Multiple prognostic studies are published yearly, providing outcome data according to different tumour or patient characteristics but they practically only report OS or DFS.

The methodological difficulties in estimating the recurrence risk in populations where interventions other than providing adjuvant chemotherapy (chiefly staging and surgery) have improved the outcomes were noted in an attempt made five years ago to produce a systematic overview of the recurrence risks reported in modern series. In the study, 25 out of 2596 randomized or observational studies published after January 2005 and enrolling patients after January 1995 provided reasonable information about the quality of the care and disease outcome [19]. Since TTR or FFR were seldom reported, conclusions were based on DFS or, occasionally RFS.

In our ambition to produce an updated systematic overview of the recurrence risks (or FFR/TTR), we relied on previous systematic overviews in identifying relevant studies. If needed, we made an update, using the same search criteria as in the overviews, as briefly described in Table 1, Table 2, Table 3, Table 4, Table 5 and Table 6.

## 3. Recurrences Risks in the Control Group of Randomized, Chiefly Colon Cancer Trials

In the “old” colon cancer trials with a surgery alone group, using OS and DFS (occasionally RFS or EFS (event-free survival) or crude recurrence rates) as endpoints, DFS in the surgery alone group after 4–5 years ranged from 44 to 62% for stages II + III together (See [77] for references to the 31 trials contributing data and Table 1 and Table 2 for the trials reporting recurrence rates/TTR). When reported separately, the corresponding figures in stage II ranged from 59 to 77% and from 35 to 44% in stage III. In a review, the EORTC group reported that 31–59% of the surgically operated patients would have a recurrence within 5 years [154], in line with the DFS reported in the trials. In the QUASAR trial [46], which mainly included stage II patients, recurrences were reported in 22%, the actuarial risk at 5 years was25%. In the recent Japanese SACURA trial [48], which only included stage II patients, recurrences were seen in 13% of the patients in the surgery alone group. Thus, recurrence risks in the order of 40–50% for stage II + III together (over 55% for stage III and about 25–30% for stage II) were seen in patients operated during the 1970–1990s. With a median age of about 62 years and a comparative short follow-up (4–5 years), most events included in DFS/RFS/EFS were recurrences and, thus, not other deaths or secondary malignancies. However, in the SACURA trial [48], where only 13% of the patients experienced a recurrence, 8% (about 40% of all events) developed a secondary malignancy, greatly influencing DFS but not RFS (Table 1).

The antibody 17-1 A was explored in a randomized study in colon cancer stage III with favourable results [89] and followed by two multicentre trials, one comparing surgery alone with surgery followed by treatment with the antibody [90,155]. The trial was negative allowing the outcome of a large group of operated stage II patients to be assessed [90]. At 7 years, the “disease-specific” DFS (i.e., TTR) was 83%, the traditional DFS was 74% (Table 2).

## 4. Recurrences Risks in the Control Group of Randomized Rectal Cancer Trials

A few randomized studies with a surgery alone group, none of which showed any significant gain from adding postoperative chemotherapy individually or collectively, allows an estimate of recurrence risks after different preoperative treatments and variable quality of the surgery, i.e., before or after the introduction of the TME procedure. In the old US trials comparing surgery alone with postoperative therapy, the risk of distant metastasis after 5 years was 33–40% in stage II and 60–70% in stage III [92,94,156], studies that resulted in adjuvant therapy becoming routine treatment at least in the US. In 4 more recent European studies [46,59,60,95] where the patients had received either preoperative short-course RT or CRT, distant recurrences were seen in 30–40% of the patients in the observation group.Five major European trials were included in an analysis with the aim of predicting the risk of the local and systemic relapse risk after CRT, i.e., to identify patients who may benefit the most from postoperative chemotherapy [103]. Thirty-one % of the patients had a distant recurrence after 5 years. This risk was marginally influenced by adjuvant chemotherapy. In yet another meta-analysis [7] based upon individual patient data from four trials, systemic recurrences were seen in 37% of patients in the control group after 5 years. In patients treated with preoperative CRT, it was 35%.

The risk of recurrence is also evaluable in trials comparing different radiation schedules or comparing conventional CRT with radiation (5×5 Gy or CRT) combined with neo-adjuvant chemotherapy (Table 3).

## 5. Recurrence Risk in Studies Exploring Different Follow-Up Routines, Comparing Open vs Laparoscopic Surgery or Using the Circumferential Mesocolic Resection (CME) Technique

Randomized or observational trials comparing different follow-up routines provide information about recurrence risks but often lack other relevant information. In three large studies including patients operated between 1998–2010 and administered adjuvant chemotherapy according to guidelines, the 4.5–5-year recurrence rates varied between 17 and 22% [110,111,112,113,114]. In six older trials, including patients operated during the 1980–90s, in which adjuvant chemotherapy was not given (except in one trial) and the quality of surgery not reported, but was probably representative of the time period, recurrences were seen more often (TTR/FFR 45–73%) in a mixture of stage I-III patients with colon and rectal cancers [104,105,106,107,108,109].

Multiple studies have since the early 1990s explored surgical techniques, some randomized comparing open with laparoscopic, conventional laparoscopic with robotic and conventional resection with more extended mesocolic resection. Multiple meta-analyses have been performed, occasionally exploring long-term outcomes including recurrence rates [119,120,121,134,135,136,157]. In the trials, approximately 20% or less have had a recurrence after up to 5 years in a mixture of stage I–III [119,125,126,127,128].

In multiple studies exploring the CME-technique [158,159], resulting in an oncologically superior specimen [160,161] versus standard colon cancer surgery, distant recurrence rates were reported in 2 studies, being about 10% after 3–4.5 years [131,132,133].

## 6. Recurrence Risks in Prognostic Studies

Numerous studies have explored the relevance of tumour-related or other factors for recurrence risks in primary CRC. It is beyond the scope of this work to review them. Furthermore, recurrence risks are seldom reported; most used parameters are OS/DFS. The outcomes are often not presented for the entire group and most data are hazard/odds ratios illustrating the differences between marker expression. Absolute recurrence risks are seldom given. The characteristics of the patients are often not well described, and they are often “convenience samples” why the representativeness is questionable.

Microsatellite instability (MSI) indicates a low risk of recurrence in primary colon cancer, at least in stage II (DFS/RFS HR 0.59 in a meta-analysis of 39 studies including 12,110 patients [162]). The absolute risk of recurrence, important for this review, was not reported. The importance of MMR-status in stage III is more unclear. In the adjuvant PETACC-8 trial, recurrences were seen in 26% of the patients operated between 2005 and 2009, 19% in MSI patients and 27% in microsatellite stable (MSS) patients (*p* = 0.02) [163]. “The need” is thus higher in patients with MSS tumours than in those approximately 15% having MSI tumours [164]. The consensus molecular subtypes (CMS) have also emerged as potentially important prognosticators with 5-year RFS rates of about 75% in CMS 1–3 and 60% in CMS 4 in 1785 patients in stages I–III from different sources, where 73% received adjuvant chemotherapy [165].

## 7. Recurrence Risk in Hospital- and Population-Based Series

### 7.1. Predominantly Colon Cancer

The MSKCC results from two time periods described above illustrate the changes seen with time [38]. In hospital-based series, recurrence rates in the order of 10–20% (/FFR/TTR rates 80–90% after 5 years) have been reported in patients operated about 10–20 years ago [55,138,140,141,142,143,144,151]. In most studies, some patients received adjuvant therapy, but several hospitals gave no adjuvant therapy [138,140,144,145] (Table 6). Similarly low recurrence risks were reported in two series of patients operated during the 1980–1990s; those centres presented information indicating that the surgical quality was “of a higher level” than at non-specialized centres [138,143]. Several studies found that in stage II, the recurrence risk at 5 years was very low in most patients whereas a minor fraction (about 10–15%) had a higher recurrence risk, motivating adjuvant chemotherapy. This has also been the conclusion of numerous prognostic studies (data not presented since chiefly only DFS/OS have been reported).

Fewer studies have reported the prognostic heterogeneity in stage III. In a German study, 1453 stage III CRC patients operated between 1978–1997, three groups with markedly different OS (no recurrence data provided) could be identified, 5-year OS was about 80% in 10% of the patients (pT1,2 N1), about 60% in 50% of the patients (*p* > T3,4 N1 or pT1,2 N2) and about 30% in the remaining 40% (pT3,4 N2) [166]. Others have also defined similar sub-grouping of stage III, including NCCN (stage IIIA–C) [4], without stating that a subgroup of stage III might not need adjuvant therapy.

Apparently fewer recurrences than historically reported after colon cancer surgery were seen in the entire Swedish population of 14,325 radically operated patients between 2007–2012 [153]. After a minimum follow-up of 5 years, 16% have had a recurrence, 11% in stage II and 29% in stage III (Table 6). When grouped according to UICC-stage, the number of risk factors (pT4, less than 12 nodes, vessel/nerve infiltration, high grade and emergency surgery) and whether adjuvant treatment was initiated (12% in stage II and 61% stage III) or not, stage II patients with 0–1 risk factor not treated with adjuvant therapy had 10% risk of recurrence, if 2 or more risk factors (19% of stage II) the risk rose to 22%. In patients where adjuvant treatment was given, marginally higher recurrence risks were seen. In stage III without risk factors, the recurrence risk was 22% without adjuvant treatment and 14% if it was initiated.

We further substantiated the apparently low recurrence risks in radically operated colon cancer patients between 2010–2015 in one Swedish region (*n* = 416). In the region, a prospective biobank initiative [167] was running, minimizing the risk of under-reporting of patients and recurrences. The results of the prospectively collected Uppsala region material were representative of the national material [153,168].

### 7.2. Rectal Cancer

In the entire Swedish and Norwegian populations of rectal cancer between 1995–2012, amounting to 29,000 Swedish and 15,500 Norwegian patients [16], preoperative RT was delivered to 61% of resected patients in Sweden versus 24%, chiefly CRT, in Norway. During the time period, local recurrence rates decreased to about 4% at 5 years from significantly higher values in Norway, whereas distant metastatic rates were identical and decreased from slightly above to slightly below 20%. Post-operative CRT was administered to some patients in Norway but not in Sweden. Adjuvant chemotherapy was rarely given in Norway and to a limited number of patients in Sweden with no change during the years.

In a hospital-based series from Finland [149], about half of the 481 rectal cancer patients received preoperative RT/CRT and 42% were administered adjuvant chemotherapy. Overall, 26% of the patients had a recurrence, local in 8% and distal in 23% (9% had both local and distant). The same quality assurance measures have not taken place in Finland as in Sweden, Norway, Denmark [101,169] and The Netherlands [147] where local recurrence rates are only a few per cent. In Denmark, only 11% of the patients developed distant metastases.

In a Chinese series of 185 rectal cancer patients curatively operated without neo-adjuvant therapy between 2006–2014 [170], 120 belonged to a high-risk group according to MRI. In these patients, distant metastases were seen in 42% and local recurrences in 10% despite postoperative CRT to 84 patients.

In recent population-based materials of rectal cancer, less than 20% of the patients develop distant metastases during follow-up, this being responsible for the majority of deaths [16,97,101,152]. In recent trials, distant recurrence rates are usually higher (30–40%), even if pre-treated with CRT, but the trials have only included patients with high-risk criteria [101,102,103]. It is, thus, more difficult to assess whether and how much distant recurrence risks have decreased in rectal cancer when compared with patients treated for colon cancer.

## 8. Recurrence Risks in Sweden during the Past Decade after Validation of Recurrence Data

### 8.1. Validation of Data

Several quality assurance programmes have been implemented in Sweden during the past decades, firstly in rectal cancer and subsequently in colon cancer [171,172,173,174]. All information is collected in the national Swedish Colorectal Cancer Registry (SCRCR), having complete coverage [172,175] and where reporting of all recurrences is mandatory once they have been diagnosed. A request is sent to the treating hospitals after 3 and 5 years. Thus, most recurrences have in all probability been recorded in the registry. According to the national care programme from 2008, updated in 2016, staging using preferably CT should be performed of the primary tumour (and MRI for rectal tumours), liver and lungs, although ultrasonography of the liver and plain chest X-ray were permitted previously. During follow-up, clinical investigation, CT of the liver and lungs and CEA are mandatory after 1 and 3 years.

The SCRCR contains demographics, staging and primary treatments with a high level of accuracy [176], but the accuracy of the M-stage at diagnosis and recurrence data has not been validated.

In the Uppsala region (375,000 inhabitants in 2018), 1707 patients were between 2010–2018 diagnosed with 1736 CRCs. The medical records of the 398 patients (23%) with registered metastases at diagnosis (265 colon cancers, 133 rectal cancers), the 201 patients in stage I–III with registered recurrences until 23 January 2020 (134, 16%, colon, 69, 13%, rectum) and the 1108 patients without a registered recurrence were re-examined.

Evidence of metastatic disease in patients registered as M1 at diagnosis was seen in all but 10 (3.6%) colon cancer patients and five (3.7%) rectal cancer patients. Reasons were chiefly misclassified liver or lung lesions, either confirmed by histology after removal of the lesion, spontaneous disappearance of the lesion or a prolonged period (at least 2 years) of lack of progression. The opposite, namely that patients had synchronous disease diagnosed at the latest at surgery, but not registered, was even more uncommon (seven (0.8%) colon cancers and one (1.4%) rectal cancer). Of the 201 recurrences, six (4.5%) colon cancer cases and one (0.3%) rectal cancer case were wrongly registered (in four cases a new colon cancer, in two cases another malignancy and in one case a haemangioma).

Nine (seven colon cancer, two rectal cancer) non-registered recurrences were diagnosed more than 3 months before data retrieval, representing under-reporting. Thus, of 722 colon cancer patients without a recurrence, seven (1%) patients had a non-registered recurrence. The corresponding figure in rectal cancer was 0.5% (2/386). It can be concluded that the number of mistakes in the register is small concerning the registration of both synchronous and metachronous metastases.

In a corrected transcript from the register performed after the completion of the validation study, 1734 cancers were diagnosed in the 1706 patients (one originally diagnosed patient without an invasive carcinoma was removed) with a primary diagnosis of CRC between 2010–2018. Twelve patients had two cancers, three had three synchronous cancers and in 10 patients a metachronous cancer developed. In the case of synchronous cancers, the least advanced was removed and in case of metachronous, the last diagnosed was removed prior to analysis for the evaluation of recurrence risks.

Few patients with colon cancer received neo-adjuvant chemotherapy (mainly those with a locally advanced non-resectable or difficult-to-resect tumour) whereas more patients received this in rectal cancer (locally advanced tumours at high risk of recurrence and included in the RAPIDO [177] or LARCT-US (ClinicalTrials.gov Identifier: NCT03729687) trials; these patients are not of interest when evaluating the need for adjuvant treatment and were excluded from the evaluations.

### 8.2. Stage-Stratified Risk of Recurrence

**Figure 1:**
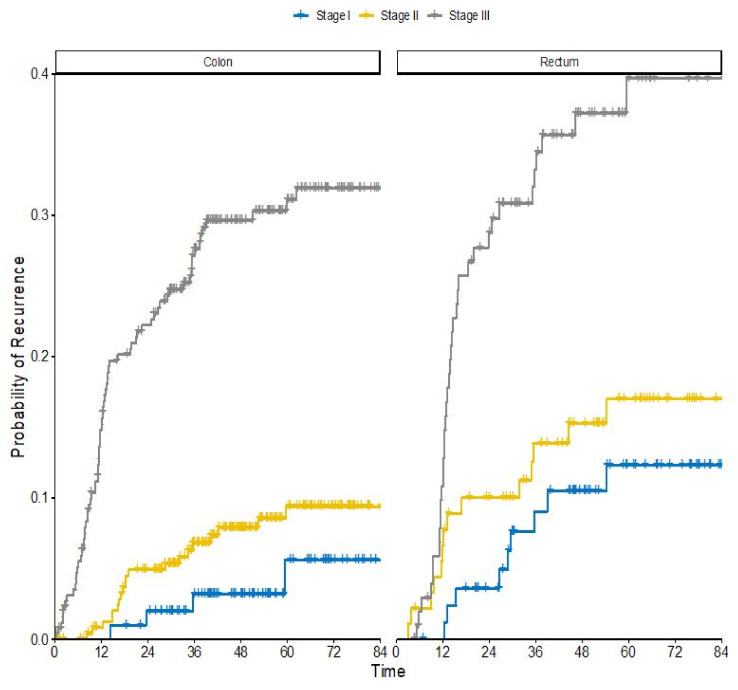
Colorectal cancer recurrence risks by tumor location and stage. Kaplan Meier event plot split by diagnosis and stage. Outcome is recurrence after radical surgery in a Swedish population-based cohort diagnosed between 2010–2017 with minimum 2 years follow-up.

Of 1212 patients with a radically operated CRC diagnosed between 2010–2018, 200 (17%) patients have had a recurrence, 16% in colon cancer and 17% in rectal cancer. In the 971 patients between 2010–2017 (ensuring a minimum follow-up of 2 years), similar recurrence numbers were seen (overall 17%, colon 16%, rectum 19%). Patient characteristics for these patients are shown in Table S1. Recurrence risks according to stage, number of risk factors and adjuvant treatment are shown in Table 7. They are for rectal cancer patients shown according to pre-treatment regimen, pathological or clinical stage, and adjuvant treatment or not. The actuarial risk at 5 years in all stages together is 17% for colon cancer and 21% for rectal cancer (Figure 1). In stages II and III, the risks are 10% and 31% in colon cancer, and 17% and 40% in rectal cancer.

Altogether, 627 patients had metastases, either synchronous (*n* = 427, 67%) or metachronous (*n* = 200, 33%). The median OS from the diagnosis of metastatic/recurrent disease was 15.6 months, i.e., considerably shorter than presently reported from clinical trials/hospital-based series [2,178], reflecting the unselected nature of the population. OS was considerably longer for those who primarily received anti-tumour treatment, mostly chemotherapy, occasionally surgery or radiotherapy (median 20.8 months) compared with those who did not (median 3.4 months).

## 9. Correlations between Time of Inclusion and Rates of Time-To-Recurrence (TTR)/Freedom from Recurrence (FFR)

**Figure 2:**
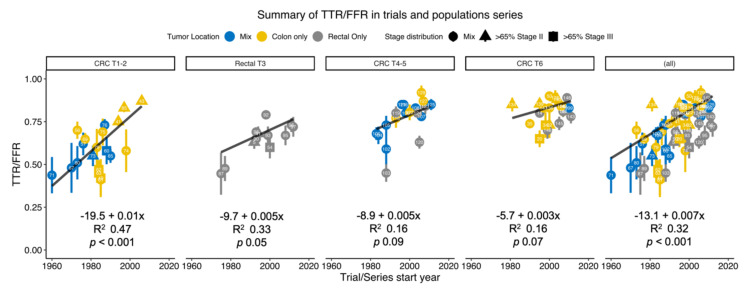
Time to recurrence (TTR) or freedom from (crude) recurrence (FFR) in surgically operated nonmetastatic colorectal cancer patients according to when the first patient was operated. Improvements with time were seen when all trials were included (right panel) and in the different types of trials as presented in the six Tables. The numbers in each filled point refer to the reference number as provided in the tables and reference list. Individual points are coloured by distribution of colon vs rectal cancer and shapes represents stage mix in each study. Linear regression for the trend, weighted by number of cases in the studies, are presented for each panel and for the total with the equation, R^2^ and *p*-value presented for each panel. In the left panel (CRC T1–2), TTR/FFR are from the untreated control group in randomized (chiefly) colon cancer trials. The best results are seen in a recent Japanese study in stage II [48], in a trial [90] including only low-risk stage II patients, and in [46], mainly including patients with stage II where the doctor was uncertain about the benefit of adjuvant therapy. In the second left panel (rectal T3) a marked improvement is seen from the two older US trials [92,94] reporting improved results after adjuvant chemotherapy/chemoradiotherapy. No apparent improvement has been observed since then. However, the trial with the best results [97] was initiated at an early stage but included most patients between 2008–2011 and included “intermediate risk” tumours as opposed to “locally advanced tumours” in most of the other trials (although most of the tumours anyhow belonged to an intermediate risk group). One of the most recent trials [177] included only patients at high risk for relapse. Also [100] included only high-risk patients. In these trials preoperative chemoradiotherapy was given to all and adjuvant chemotherapy to some. Two of the older trials [95,96] had worse results despite including less advanced (most cT3 and not cT4) tumours. In the middle chart (CRC T4–5), being a systematic review of all randomized surveillance and laparoscopic trials, a clear improvement with time is seen. However, adjuvant therapy was provided to more patients in the recent trials than in the older trials, explaining some of the improvement. The two studies with the best results used the circumferential mesorectal technique, CME, potentially explaining few recurrences. In the second chart to the right (CRC T6), including recent patient series, the results are apparently better than in the older trials. Few recurrences were seen in an early trial from Erlangen [138], where the surgical quality was “at a high level”. No adjuvant therapy was provided, further emphasizing the good results. Besides this trial, there still appears to be an improvement with time, but the studies are heterogenous and many factors may lie behind this improvement.

Rates of TTR/FFR in the different trials shown in Table 1, Table 2, Table 3, Table 4, Table 5, Table 6 and Table 7 according to the start of patient inclusion are shown in Figure 2 separately for the adjuvant trials with a untreated control group (Table 1 and Table 2 for predominantly colon cancer and Table 3 for rectal cancer), for the surveillance and surgical technique studies (Table 4 and Table 5), the hospital- and population-based series (Table 6 including the Swedish data presented in Table 7) and for all studies together. Although the studies within each group are very heterogeneous, significant correlations were found. In all studies together, the improvement was 0.7% (*p* < 0.001) in linear regression adjusted for case numbers. When further adjusted for tumour location, stage distribution, the improvement was 0.54% (*p* < 0.001). The figure legend describes the position of individual trials with respect to inclusion times and stage distributions. Details of the extent of adjuvant therapy provided are shown in the tables.

## 10. Discussion

This overview has raised a practically important question for many doctors/patients worldwide but, for methodological reasons, this question is impossible to give a precise answer to. Even if an accurate estimate of the magnitude of improvement cannot be given and may not even be needed, it is evident that the risk of recurrence after radical CRC surgery and, thus, the need for adjuvant chemotherapy, is less today than in the past. This should influence how patients are informed and whether and which adjuvant therapy is indicated. General guidelines by international organizations, such as NCCN and ESMO [4,9,40] do, in our opinion, not properly consider this continuous development, that most probably has not yet come to an end. Only few studies, including one from MSKCC have recently pointed to this dilemma [34]. This is positive news for many patients since fewer will experience a recurrence, but if not corrected will result in overtreatment of more patients than was the case in the past.

It is extremely difficult to initiate a new generation of randomized adjuvant trials, at least in colon cancer, having a surgery only group. It is likewise similarly difficult in rectal cancer; the negative experience of trying to run such trials by several collaborative groups during recent years speaks against any success [59,60]. Even if those recent randomized trials did not show sufficient gains, adjuvant chemotherapy is frequently given according to principles in colon cancer, further emphasizing the difficulties [4,9,40,179]. Thus, further analyses of the results of unselected populations are needed; this is, however, for obvious reasons hampered by the selective provision of adjuvant chemotherapy. Several measures could, however, be taken to improve the relevance of population-based studies, one of which is the reporting of recurrences.

In order to overcome the obstacles of poor tumour recurrence registration in population registers, a US study stated that if intense support is given to such registries, the recurrences can be properly captured and more generalizable results be obtained [180]. In Sweden, it is the duty of individual hospitals to feed the quality registries with proper and complete data, but the registries are voluntary and thus rely on whether the departments can create enough resources to complete data. However, the incentive for all departments to have their own data as complete as possible is strong since anonymized key data per hospital is officially released yearly with rankings. Furthermore, the quality registries are excellent resources for research [181]. Many hospitals participate in research projects, and none wants to belong to a group having incomplete and poor data. Our extensive validation in one region in Sweden, detecting extremely few mistakes, tells that the Swedish Registry is characterized by a high degree of validity.

Since follow-up routines in Sweden are not intensive, it is possible that a patient dying of another cause may have developed a recurrence, if death had not intervened. There is thus, always a possibility that the number of patients with subclinical disease, requiring adjuvant therapy, is larger than the number with detected recurrences, but the extent of this is probably small. Even if adjuvant chemotherapy was selectively provided according to guidelines, this does not prevent conclusions to be made, at least not in stage II where the use was limited. Additionally, since much evidence tells us that recurrence risks are independent of age, many elderly patients not treated may provide relevant information. Intercurrent deaths are common among the elderly, but most recurrences come early or within a few years and the survival prospects of older adults having survived major surgery are longer.

## 11. What Were the Risk of Recurrence a Few Decades Ago and What Are They Presently If Adjuvant Treatment Is Not Given?

### 11.1. Colon Cancer

In the randomized trials comparing surgery alone with surgery and adjuvant chemotherapy, TTR/FFR varied between 41 and 60% and the RFS/DFS between 44 and 67% in stages II + III together in the surgery alone group after about 5 years. In stage II, RFS/DFS varied between 59 and 79% and in stage III between 29 and 44% (since median age was 62 years and follow-up comparatively short, most events were caused by recurrences). Further, a few more recurrences will occur with a longer follow-up, indicating that subclinical disease after surgery performed during the 1970–90s was present in about 40–50% of stage II + III patients (Figure 2), in 30–35% of stage II patients and in 60–70% in stage III, whether included in a clinical trial or not. In a few trials exploring experimental treatments during the same time period, recurrence risks were 30–40% overall, 60% in stage III but only 10% in one trial including only low-risk stage II patients (pT4 bN0-cases were excluded) randomized between placebo or an ineffective treatment [90], The need for adjuvant chemotherapy was, thus, previously quite substantial although it was possible to identify groups with limited need.

The two retrospective subgroup analyses of patients included in the ACCENT database, revealing better results in more recent compared with the older trials that could be ascribed to fewer recurrences, did not quantify the magnitude of the difference [31,32]. When an old nomogram (by MSKCC) was validated, 7–8 percentage points fewer recurrences in both stage II and III were seen (Table 6) [33].

In our overview of trials comparing two different surveillance strategies, the recurrence risks varied according to when the patients were operated. In 5 trials including patients between 1983–1996, recurrence risks after 5 years ranged from 31 to 55% [105,106,107,108]. It is likely that no patients received adjuvant chemotherapy in those trials. In a trial including patients between 1997 and 2001, the recurrence risk was 27% at 4 years, however, it was stated that all patients should have been administered adjuvant treatment, this possibly being the reason for the slightly better results. In the most recent trials [110,111,112,113,114], the recurrence rates have varied been between 17 and 20%, but adjuvant chemotherapy was selectively provided. In recent trials comparing surgical details, recurrence risks have been even lower or overall, about 15%, however, with a shorter follow-up. The improvements with time in these “surgical” trials can, thus, be the result of more recurrence-reducing adjuvant chemotherapy but also that recurrences have become less frequent. However, a few hospital-based series, where no adjuvant treatment was provided and where statements about high quality in the care were made, similarly report recurrence rates in the order of 10–15% for stage II patients [140,144,145,146,166]. This was also the case in the surgery alone group in the recent randomized Japanese trial [48]. With further support from the Swedish population-based data ([153] and further presented here after validation of the register data, Table 7), it is reasonable to conclude that recurrence rates overall are in the order of 15–20%, about 10–12% for stage II (without adjuvant chemotherapy) and about 25–30% for stage III. The estimate for stage III is uncertain due to the paucity of studies and that adjuvant chemotherapy has been administered to many patients.

### 11.2. Rectal Cancer

Without doubt, the risk of local failure has decreased during the past several decades down to less than about 5% at dedicated hospitals and in national populations [2,16,147,148,173,182]. Better staging, better surgery and appropriate use of pre-operative treatments are responsible. In the early trials, systemic recurrences were if anything a few percent higher in rectal cancer than in colon cancer, or overall, up to 50%. Similar to the situation in colon cancer, these risks have decreased, but remain around 30–40% in patients with “locally advanced rectal cancer” included in the radiotherapy trials. In populations and in the most recent generation of radiotherapy trials, they remain around 20% in patients with intermediate risk tumours (bad group) and around 30% if locally advanced (bad/ugly group) despite being treated with pre-operative CRT (about 50 Gy with a fluoropyrimidine), indicative of the present need for adjuvant therapy on this level. In these patients, multiple trials have not shown any clear beneficial effect of adjuvant chemotherapy. During ASCO 2020, two randomized trials reported fewer systemic recurrences using neo-adjuvant chemotherapy [101,102]. Most patients with locally advanced tumours may thus in the future receive total neoadjuvant therapy (TNT), although some patients will probably have a need for additional post-operative treatment, like those where staging MRI misinterpreted the findings and where the response to the TNT was poor. The chances of beneficial effects from conventional chemotherapy will probably be minimal in the latter group.

### 11.3. Methodological Considerations

Any summary of heterogeneous data collected during different time periods, as provided in Figure 2, can be subject to bias. Despite the great heterogeneity, particularly with provision of adjuvant therapy in the recent series, the strong correlations including the visually strong impression are probably true but overestimated. We have considered several potential errors in the legend to Figure 2. The strong correlation between response rates to first line chemotherapy and liver resection rates in metastatic CRC confined to the liver, reported 15 years ago, identified an important correlation, although it was exaggerated (Figure 1 in [183]).

## 12. Conclusions

Interstudy comparisons, particularly if the trials were performed decades apart, cannot allow firm conclusions, but most evidence tells us that recurrence risks are substantially lower today than they were when the trials showing the benefit from adjuvant chemotherapy were performed, at least in the case of colon cancer. It is, thus, no longer appropriate to treat patients based upon old data as still recommended in most guidelines. It is not possible to give an exact figure of how large the improvement is, although the numbers seen in the Swedish material (see Table 7), where not a single patient or recurrence was missed, together with the recent surveillance studies (Table 4), the studies exploring surgical techniques (Table 5) and several surgical and population-based series (Table 6) give a good indication of what can be achieved at centres/in countries having an interest in the quality of the CRC care. Many patients with stage II that previously were at sufficient risk of recurrence (1 risk factor) probably have such a limited recurrence risk (<10%) that adjuvant treatment is not motivated. Nevertheless, some (maybe 20%) stage II patients (presence of the high-risk factors pT4 or <12 lymph nodes or 2 or more other risk factors) [3] still may have a sufficiently high risk (about 15–20%) to motivate additional treatment, although not necessarily with oxaliplatin. Conversely, some (maybe 20–25%) stage III patients have such a low recurrence risk (about 20%) that the addition of oxaliplatin can be questioned. It should be noted that not only the recurrence risk is important when deciding whether treatment should be recommended or not but also patient related factors and, above all and not reviewed here, the likelihood that the treatment will eradicate tumour cells.

## Figures and Tables

**Table 1:** Time to recurrence rate/freedom from recurrence and recurrence-free and disease-free survival in adjuvant predominantly colon cancer trials with a control group where systemic chemotherapy was given in the experimental group(s).

Trial/Reference	Inclusion Years	Number Control Pts/Total Number Pts	Number of Control Patients in Stage I/II/III	Colon/Rectum	Proportion Receiving ACT	Follow-Up Time (Years)	FFR/TTR	RFS/EFS	DFS	Comments
VASOG [61]	1973–1979	318/645	0/182/136	NR, majority colon cancer	0	5	70%			
Hafström et al. [62]	1976–1978	205/421	0/98/75	141/64	0		II: 75% III: 45%			
NCCTG [63]	?-1985	135/401	0/49/86	127/8	0	5		All: 44% II: 59%, III: 35%,		
Dahl et al. [64]	1993–1996	206/412	0/126/80	146/65	0	5			All: 63% III: 37%	
QUASAR uncertain [46]	1994–2003	1143/2291	0/1073/70	100/0	0	5	75%			Stage II at 10 years projected TTR 71%
O’Connell et al. [65]	1988–1989	151/309	0/27/124	124/27	0	5	58%			
Francini et al. [66]	1985–1990	118/239	0/60/58	100/0	0	5	41%			
Moertel et al. [67]	1984–1987	315/1296	0/?/315	100/0	0	3.5	All: 47% II: 67%			
Moertel et al. [68]	1984–1987		0/0/929	100/0	0	5	45%			At 8 years projected FFR 40%
GIVIO-SITAC 01 [69]	1989-?	446/869	0/228/218	100/0	0	5		54%		
Taal et al. [70]	1990–1996	575/1029	0/235/280	280/235	0	5	All: 55% III: 46%	49%		76% of the recurrences were distant
SWOG, Panettiere et al. [71]	1977–1988	94/317	NR	80/14	0	7		44%	44%	
Windle et al. [72]	NR–1970 s	45/141	NR	26/19	0	5	48%			
IMPACT-1 [73]	1982-?	757/1493	0/423/334	100/0	0	3		All: 62% II: 76% III: 44%		FFCD, GIVIO, NCIC-CGT trials
NSABP-C-01–05, Wilkinson et al. [74]	1977–1994	693/2966	Stage II, 51%	100/0	0	5	II: 77%, III: 52%	60%	50%	Pooled data from 5 trials. FFR at 10 years stage II 73%, stage III 44%. Less than half <12 nodes
IMPACT-2 [75]	1982-?	509/1116	0/509/0	100/0	0	5		73%		FFCD, GIVIO, NCIC-CGT, NCCTG Intergroup trials
Matsuda et al. [48]	2006–2010	997/1982	0/997/0	100/0	0	5	87%	85%	78%	78% 12+ nodes, median 19. The worse DFS than RFS (and FFR) is mainly caused by secondary malignancies
Li and Ross [76]	1960–1965	84/213	53/41	NR	0	5	II: 59%, III: 24%			Historical controls

These trials were identified in a meta-analysis/systematic overview [77] and only one further study using a surgery only group was identified [48]. The key publications for all trials were scrutinized to find information of recurrence rates (or TTR) and not only DFS or OS as presented in the overview. Abbreviations: DFS = disease-free survival, OS = overall survival, RFS = recurrence-free survival, EFS = event-free survival, TTR = time to recurrence, FFR = freedom from recurrence (100-crude recurrence rate in % as provided in the articles), ACT = adjuvant chemotherapy, NR or ? = not reported. In the individual trials, RFS/DFS is only presented if FFR/TTR was not available.

**Table 2:** Time to recurrence rate/freedom from recurrence and disease-free survival in adjuvant colorectal cancer trials with a control group where regional chemotherapy or miscellaneous treatments were administered in the experimental group(s).

Trial/Reference	Inclusion Dates	Number Control Pts/Total Number Pts	Number of Patients in Stage I/II/III	Colon/ Rectum	Proportion Receiving ACT	Follow-Up Time (Years)	FFR/TTR	DFS	Comments
SAKK et al. [78]	1981–1987	266/533	0/157/79	161/92	0	5	55%	All: 48% II: 63% III: 29%	
Scheithauer et al. [79]	1998–1990	60/121	0/31/29	60/0	0	4.5	58%		Intraperitoneal and intravenous
Vaillant et al. [80]	1986–1991	134/267	0/77/57	134/0	0	5	69%	All: 62% II: 69%	
Rougier et al. [81]	1987–1993	619/1235	113/262/217	367/232	0	5	73%	65%	
Wolmark et al./NSABP C02 [82]	1984–1988	459/901	114/202/140	459/0	0	4		64%	
AXIS [83]	1989–1987	1792/3583	186/707/514	1018/774	0	5		55%	DFS colon 57%, rectum 51%, if curative resection 64%
EORTC-GITCCG [84]	1983–1987	79/235	6/41/23	72/0	0	9	60%	48%	
Lawrence et al. [85]	1973–1975	101/203	?/64/37	62/39	0	5	51%		Stage I, II not separated
Wereldsma et al. [86]	1981–1984	102/372	NR	58/44	0	3.7		58%	Rotterdam trial, only OS data
Irvin et al. [87]	NR	65/128	5/29/33	38/29	0	5	66%		Only liver metastases reported
Hanna et al. [88]	1980-?	159/233	NR	NR	0	5		68%	Vaccination
Hanna et al. [88]	1980-?	217/324	NR	NR	0	5		62%	Vaccination, unclear reporting
Riethmuller et al. [89]	1985–1990	76/189	0/0/76	100/0	0	5	42%	38%	Treatment with 17-1 A antibody
CALGB 9581 [90]	1997–2002	873/1738	0/873/0	100/0	0	7	83 (81–85)%	74 (72–76)%	Surgery +/− 17-1 A antibody. Note the difference in TTR (designated disease-specific DFS) and DFS at 7 years

These trials were identified in two meta-analysis/systematic overviews [77,91] and no further studies using a surgery only group were identified when the same search strategies were used as in [77,91]. The key publications for all trials were scrutinized to find information of recurrence rates and not only DFS or OS as presented in the meta-analyses. Abbreviations: DFS = disease-free survival, OS = overall survival, TTR = time to recurrence, FFR = freedom from recurrence (100-recurrence rate in % as provided in the articles), ACT = adjuvant chemotherapy, NR or ? = not reported.

**Table 3:** Time to recurrence rate/freedom from recurrence and recurrence-free or disease-free survival in adjuvant trials in rectal cancer with a surgery alone group and where systemic chemotherapy was provided in the experimental group.

Trial/Reference	Inclusion Dates	Number Control Pts/Total Number Pts	Number of Patients in Stage I/II/III	Proportion Receiving ACT	Preoperative Treatment	Follow-Up Time (Years)	TTR/FFR	RFS/DFS	Comments
GITSG [92]	1975–1980	62/227	0/21/37	0	None	5	II: 67% III: 32%		Before TME
NSABP R01 [93]	1977–1986	179/555	0/67/109	0	None	5		29%	Before TME, DM risks given in [94]
Gunderson et al., 5 US trials pooled [94]	1977–1986	179/3791	0/67/109	0	None	5	II: ~60% III: ~40%		Present DM rates of the NSABP-trial, 40% pT1–2 N+, 60% pT3 N1, 34% pT3 N0, 59% pT3 N2
QUASAR uncertain [46]	1994–2003	474/948	0/407/67	0	6% neo-RT (8% adj-RT)	5	68%		Before TME, projected 5 year
EORTC 22921 [95]	1993–2003	505/1011	NR	0	RT or CRT	5	65%	52%	90% T3, 10% T4. 34% DM overall
Gerard et al., FFCD [96]	1993–2003	0/742	87% T3, 13% T4	70%	RT or CRT	5		57%	Before TME, LR 17% RT vs 8% CRT, adjuvant chemo planned both groups
PROCTOR/SCRIPT [59]	2000–2013	221/437	0/32/189	0	5 × 5 or CRT	5	60%		Systemic recurrence 39%, local 8%
Chronicle [60]	2004–2008	59/113	31/28	0	CRT	3		73%	
Stockholm III [97]	1998–2013	920	NR	About 15%	5 × 5 direct or delayed surgery, RT 2 × 25	5	projected 79%	65%	Intermediate risk tumors. ACT only recorded in patients included from 2007. ypTN I/II/III/IV/X = 271/250/278/25/11
Bujko et al., Polish I trial [98,99]	1999–2002	316	170/113	NR	5 × 5 direct surgery or CRT	4	67%	57%	Locally advanced, low-lying
Polish II trial [100]	2008–2014	254/515	NR	39%	CRT	8	67%	41%	TNT= FF-DM
RAPIDO [101]	2011–2016	452/920		42%	CRT	3	73%	70%	Locally advanced, ugly tumours, TNT provided in experimental group, RFS/DFS = DrTF, TTR/FFR = FF-DM
PRODIGE 23 [102]	2012–2017	230/461		69%	CRT	3	72%	69%	TNT in experimental group, RFS/DFS = FF-DM
Valentini et al., Five European trials pooled [103]	1993–2003	1209/2795	1879/833	56%	RT/CRT	5	Distant all 69%, ypN0 79%, ypN1–2 48%, local all 87%		Created a nomogram. ACT limited effect. Few events after 5 to 10 years (distant all from 69% to 66%)
Bregoum et al. [7]	1992–2013	598/1196	207/391	0	5 × 5 or CRT	5	63%		Meta-analysis 4 trials, TME, FF-DM

The old trials were identified in one meta-analysis/systematic overview [77] and the more recent ones in three overviews/meta-analysis [7,8,103] and four further studies where the recurrence risk could be described after preoperative RT [97] or CRT [98,99,100,101] were identified. The key publications for all trials were scrutinized to find information of recurrence rates and not only DFS or OS as mostly presented in the overviews. Abbreviations: DFS = disease-free survival, OS = overall survival, RFS = recurrence-free survival, EFS = event-free survival, TTR = time to recurrence, FFR = freedom from recurrence (100-recurrence rate in % as provided in the articles), TME = total mesorectal excision, DM = distant metastasis, RT = radiotherapy, CRT = chemoradiotherapy to 46–50 Gy, 5 × 5 = 5 times 5 Gy radiotherapy in one week, FF-DM = freedom from distant metastasis, DrTF = disease-related treatment failure, ACT = adjuvant chemotherapy, TNT total neoadjuvant treatment evaluated in the experimental arm.

**Table 4:** Time to recurrence rate/freedom from recurrence and disease-free survival in studies comparing different follow-up routines in colorectal cancer.

Trial/Reference	Inclusion Years	Total Number of Pts	Number of Patients in Stage I/II/III	Colon/Rectum	Proportion Receiving ACT	Follow-Up Time (Years)	TTR/FFR	DFS	Comments
Kjeldsen et al. [104]	1983–1994	597	138/293/166	313/284	0	5	68%		Recurrence risk 13% stage I, 20% stage II, 48% stage III, slightly higher in rectum than in colon
Ohlsson et al. [105]	NR	107			0	5	67%		Limited information provided
Mäkelä et al. [106]	1988–1990	106	28/48/30	75/31	0	5	59%		Recurrence risk 36% stage I, 38% stage II, 50% stage III
Secco et al. [107]	1988–1996	358	?/201/137	0/358	0	5	45%		Did not separate stage I + II
Schoemaker et al. [108]	1984–1990	325	71/153/101	238/87	0	5	67%		Median number of nodes = 7
Rodriguez-Moranta et al. [109]	1988–2001	259	0/157/102	194/65	100%	4	73%		
GILDA [110]	1998–2006	1228	0/617/611	933/295	85%	5	80%	75–82%	DFS about 73% at 8 years
COLOFOL [111]	2006–2010	2555	0/1352/1203	1671/884	47%	5	78%	NR	5-year cancer-specific survival stage II 93%, stage III 84%
FACS [112,113,114]	2003–2009	1202	254/553/354	843/359	41%	4.4	83%		Recurrence risk 16% colon, 24% rectum, 9% stage I, 16% stage II, 27% stage III

The trials were included in a systematic review published in 2015 [115]. Three trials [116,117,118] did not provide any meaningful recurrence data. Using the same search criteria, one additional study [111] was found. Abbreviations: TTR/FFR: time to tumour recurrence/freedom from recurrence (100-recurrence rate in % as provided in the articles), DFS = disease-free survival, ACT= adjuvant chemotherapy, NR or ? = not reported.

**Table 5:** Time to recurrence rate/freedom from recurrence and disease-free survival in studies comparing open vs laparoscopic surgery or studies where patients were operated with a circumferential mesocolic resection (CME).

Trial/Reference	Inclusion Years	Total Number of Pts	Number of Patients in Stage I/II/III	Colon/Rectum	Proportion Receiving ACT	Follow-Up Time (Years)	TTR/FFR	DFS	Comments
Cochrane et al. [119]	NR	3346	NR	2518/828	NR	NR	86–94%		Meta-analysis 7 trials, short follow-up, many likely adjuvant/neo-adjuvant treatment
Liang et al [120]	1997–2006	2474	NR	NR	NR	At least 2	85%		Meta-analysis 10 trials, local recurrence 5%, distant 11%
Ng et al. [121]	NR	169,236	NR	NR	NR	NR	86%		Meta-analysis 73 trials, 6 RCTs
Lacy et al. [122]	1993–1998	219	45/90/73	100/0	58%	7.5	77%		Majority locoregional recurrences
Leung et al. [123]	1993–2002	403	59/145/133	0/100	21% (II: 12%, III: 55%)	5	80%	77%	Distant metastasis in 17%
Tan et al. [124]	2005–2009	633	119/166/246	0/100	34%	5	63%	65%	No RCT, median 14 lgll, 5% preop RT/CT
CLASSIC [125]	1996–2002	794	132/281/288	413/381	28%	3	Distant 85%, local 92%	67%	C: 12%, R: 17%, LR C: 7%, R:10%
Liang et al. [126]	2000–2004	286	0/132/137	100/0	NR	3	II: 85% III: 76%		Additionally, 4% had recurrences after 3 years
ACOSOG Z6051 [127]	2008–2013	242	2/99/141	0/100	46%	4	84%		preoperative CRT 86%
COLOR II [128]	2004–2010	1044	338/271/358	0/100	NR	3	80%	73%	preop RT/CRT 60%
ROLARR, Jayne et al. [129,130]	2011–2014	471	132/296/175	NR	47%	3	85%	76%	Robotic vs conv laparoscopy, 46% preoperative treatment
Storli et al. [131,132]	2007–2010	251	60/117/74	100/0	NR	3	87%	77%	CME, 83% 12+ nodes, TTR stage I 95%, stage II 93%, stage III 70%
Shin et al. [133]	2006–2009	168	0/87/81	100/0	54%	5	92%	88%	CME, 94% 12+ nodes, RR 5% stage II, 12% stage III

Multiple meta-analyses have been identified exploring various aspects of the outcomes after open vs laparoscopic surgery, whether performed conventionally or more lately as robotic surgery [120,121,134,135,136,137]. The far majority have only reported short-term outcomes. The studies included above are the largest trials reporting reasonably long follow-up times and risk of recurrence. No additional trials were identified. Abbreviations: TTR/FFR: time to tumor recurrence/freedom from recurrence (100-recurrence rate in % as provided in the articles), DFS = disease-free survival, RR: recurrence risk, CME = circumferential mesocolic excision, RCT = randomized clinical trial, NR = not reported, RT = radiotherapy, CRT = chemoradiotherapy, ACT = adjuvant chemotherapy, LR = local recurrence, C = colon cancer, R = rectal cancer, lgll = lymph nodes.

**Table 6:** Time to recurrence rate/freedom from recurrence, recurrence- and disease-free survival in surgical or population-based series of colon or rectal cancer.

Trial/First Author	Inclusion Dates	Total Number Pts	Number of Patients in Stage I/II/III	Colon/ Rectum	Proportion Receiving ACT	Follow-Up Time (Years)	TTR/FFR	RFS/ EFS	DFS	Comments
Konishi et al., MSKCC early [38]	1990–2000	1320	421/520/379	100/0	II: 14% III: 85%	5	II: 81% III: 64%			61% 12+ nodes, created a nomogram
Konishi et al., MSKCC late [38]	2007–2014	1095	286/425/384	100/0	II: 25%, III: 89%	5	II: 89%; III: 72%	85%		97% 12+ nodes, created a new nomogram because of better results
Collins et al. [35]	2000–2005	134	19/90/25	100/0	46%	5	73%	48%		Validated the early MSK nomogram, projected
Kazem et al. [37]	1998–2003	138	0/10/128	100/0	30%	5	73%			Validated the early MSK nomogram
Merkel and Erlangen [138]	1981–1997	305	0/305/0	100/0	0	5	85%			Well documented quality of the surgery. A small high-risk group identified
Touchefeu et al. [139]	2003–2009	195	0/195/0	100/0	17%	3	89%		83%	93% 12+ nodes, DFS projected
Yamanaka et al. [140]	2000–2005	1487	0/1010/564	100/0	0	5	All 83%, II: 89%, III: 74%			12 hospitals
Lavanchy et al. [141]	2002–2013	475	94/118/98	334/141	29%	5		88%		Unclear if RFS or DFS, 165 pts stage IV
Wanis et al. [142]	2006–2015	1180	233/503/444	100/0	31%	5	84%		83%	If emergency surgery TTR:71%
Tsikitis and Mayo [143]	1995–2007	1136	0/871/265	100/0	II: 20%, III: 72%	5	II: 90%, III: 70%			Mean 17 nodes sampled. Intensive follow-up
Amri et al., MGH [144]	2004–2011	313	0/313/0	100/0	0	5	88%			TTR 7% 0–1 risk factor (CEA, high grade, PNI, EMVI, *n* = 124), 18% if 2–3 risk factors, (*n* = 50), 25% if all 4 (8 patients), 90% 12+ nodes
Gertler et al. [145]	1982–2006	492	0/492/0	100/0	0	10		84%		85% had RFS 87%, 15% RFS 75%, 83% 12+ nodes. Most patients operated after 1996
Kumar et al. [146]	1999–2008	1697	0/1697/0	100/0	Low risk: 12%, High risk: 29%	3				High-risk group (*n* = 1286), RFS 79 vs 80% if AC/no AC, Low risk-group (*n* = 411), 84% RFS if AC, 93% if no AC. No overall data presented
Tersteeg et al. [147]	2011–2016	407	286/121	0/100	NR	2	proj 78%			2% LR, present early results after changed guidelines for RT/CRT
Ruppert et al., OCUM [148]	2007–2016	545	122/125/298	0/100		5	81%		72%	41% (*n* = 174) preop CRT. 5-year DM 19%, 3% LR, 97% had mesorectal plane excision
Rasanen et al. [149]	2005–2011	481	116/129/167	0/100	42%	5	74%			About half had preop CRT. DM at 5 years in 18% stage O-II, 30% stage III
Tan et al. [150]	1999–2007	326	71/106/149	0/100	34%	10	All: 70%			LR: 8%, DM 22% (42% if ACT, 12% if no ACT), 99% of recurrences within 5 years
Ishihara et al. [151]	1997–2006	5664	2877/2787	100/0	38%	5	All: 83% II: 90% III: 76%	83%		22 hospitals, right-sided 84%, left-sided 81%
Chapuis et al. [23]	1995–2010	363	0/0/363	100/0	56%	5	All: 65%			CME, competing risk analysis, no difference if ACT or not
Mroczkowski et al. [55]	2000–2004	15,096	5451/9645/8616	100/0	NR	5	90%			80% 12+ nodes, only 68% adequate follow-up, questioning the recurrence data
Poulsen et al. [152]	2009–2010	1633	524/553/502	0/100	NR	5	89%			LR 4%, 11% systemic recurrences, 54 pts stage IV, 479 (29%) had preop CRT
Osterman et al. [153]	2007–2012	14,325	2,730/6,314/5,201	100/0	II: 12%, III: 61%	5	All: 84% II: 89% III: 71%			Stage II 0–1 risk factor (pT4, <12 nodes, high grade, emergency surgery, vessel/nerve infiltration.) no ACT 90%, 2+ risk factors 78%. Stage III 0 risk factor 78%. 82% 12+ nodes
Glimelius et al. [16]	1995–2012	28,962	NR	0/100	NR, limited	5	80%			LR down to 4% in both countries from higher values in Norway, DM decreased from 22% to 18% during the time period in both countries
Uppsala, Sweden (present article)	2010–2017	1212	172/381/410	806/406	II: 19%, III: 62%	5	83%			TTR 84% colon, 83% rectum, for further details, see Table 7.

The same search strategies as used in a previous similar systematic overview [19] evaluating “modern” recurrence risks is colon cancer patients were utilized for this overview. We did not exclude articles that did not present stage-specific results. Totally 25,588 articles were identified, of which the above contained relevant information. In the previous overview, it was reported that patients operated between 1995–2008 and not treated with adjuvant chemotherapy had a DFS (TTR was not reported adequately) of 81% in stage II (*n* = 2250) and 49% in stage III (*n* = 312). If adjuvant chemotherapy was provided, DFS was 79% vs 64%. Few of the 37 evaluated studies reported the quality of the care [19]. Abbreviations: TTR/FFR = time to tumour recurrence/freedom from recurrence (100-recurrence rate in % as provided in the articles), RFS/EFS = recurrence-free/event-free survival, DFS = disease-free survival, LR = local recurrence, DM = distant metastasis, RR = recurrence risk, ACT = adjuvant chemotherapy.

**Table 7:** Recurrence rates at 3 and 5 years after radical surgery in a Swedish population-based patient cohort diagnosed between 2010–2017 according to whether adjuvant therapy was initiated or not.

Colon Cancer	**Stage**	**Risk Factors**	**No Adjuvant Treatment**			**Adjuvant Treatment**		
				**Recurrence Rate**			**Recurrence Rate**	
			***n***	**3 Year**	**5 Year**	***n***	**3 Year**	**5 Year**
	Stage II	*0–1*	182	3%	6%	27	12%	18%
		≥2	25	23%	23%	23	14%	14%
	Stage III	0	28	22%	22%	44	9%	12%
		1	30	36%	36%	42	15%	15%
		≥2	42	38%	55%	75	39%	43%
Rectal Cancer	**Primary Treatment**	**Stage**	**No Adjuvant Treatment**			**Adjuvant Treatment**		
		**pStage**		**Recurrence Rate**			**Recurrence Rate**	
			***n***	**3 Year**	**5 Year**	***n***	**3 Year**	**5 Year**
	Direct surgery or scRT without delay to surgery	I	46	9%	12%	0	-	-
		II	42	5%	5%	1	0%	0%
		III	18	47%	55%	35	23%	28%
		**cStage**						
	scRT with delay or scRT and chemotherapy or CRT	I	4	0%	50%	0	-	-
		II	10	10%	10%	2	50%	-
		III	108	22%	28%	32	16%	16%
	before surgery	-	-	-	-	-	-	-
		-	-	-	-	-	-	-

Abbreviations: scRT= short-course radiotherapy. CRT= chemoradiotherapy, *n*= number of patients. pStage= pathological stage, cStage, clinical stage using pelvic MRI. For risk factors, see Table S1.

## Supplementary Materials

The following are available online at https://www.mdpi.com/2072-6694/12/11/3308/s1, Table S1: Demographics of non-metastatic patients in a population-based Swedish Colorectal Cancer cohort diagnosed between 2010–2017.
