# Supplementary material for: Recurrence Risk after Radical Colorectal Cancer Surgery—Less Than before, But How High Is It?
Source: Cancers (Basel). 2020 Nov 9;12(11):3308. doi: 10.3390/cancers12113308 (PMC7696064; doi:10.3390/cancers12113308)
Supplement: Supplementary file 1 [file cancers-12-03308-s001.pdf]

Supplementary Material: Recurrence Risk After Radical Colorectal Cancer Surgery—Less Than Before, But How High Is It?

Erik Osterman, Klara Hammarström, Israa Imam, Emerik Österlund, Tobias Sjöblom and Bengt Glimelius

Table 1. Demographics of non-metastatic patients in a population-based Swedish Colorectal Cancer cohort diagnosed between 2010-2017.

Variable	Characteristics	Total	Colon	Rectum
Sex	Male	531 (55%)	334 (52%)	197 (60%)
	Female	440 (45%)	310 (48%)	130 (40%)
Age	Mean (SD)	70 (±12)	71 (±12)	67 (± 11)
Tumour location	Right	359 (37%)	359 (56%)	
	Left	284 (29%)	284 (44%)	
	Rectal	327 (34%)		327 (100%)
	Missing info	1 (0%)	1 (0%)	
Surgery	Elective	864 (89%)	539 (84%)	325 (99%)
	Emergency	107 (11%)	105 (16%)	2 (1%)
Found nodes	Mean (SD)	22 (± 12)	24 (± 13)	19 (± 11)
	Missing information	44 (5%)	26 (4%)	18 (6%)
Risk factors*	0	499 (51%)	311 (48%)	188 (57%)
	1	274 (28%)	166 (26%)	108 (33%)
	2+	198 (20%)	167 (26%)	31 (9%)
	Adjuvant treatment			
Stage I	No	140 (94%)	103 (99%)	37 (82%)
	Yes	9 (6%)	1 (1%)	8 (18%)
Stage II	No	266 (83%)	207 (73%)	59 (94%)
	Monotherapy	28 (9%)	28 (18%)	0 (0%)
	Combination therapy	26 (8%)	22 (9%)	4 (6%)
Stage III	No	133 (41%)	100 (38%)	33 (25%)
	Monotherapy	66 (20%)	66 (25%)	0 (0%)
	Combination therapy	126 (39%)	95 (36%)	31 (25%)
Recurrence	No	808 (83%)	544 (84%)	264 (81%)
	Yes	163 (17%)	100 (16%)	63 (19%)
Local recurrence	No	943 (97%)	628 (98%)	315 (96%)
	Yes	28 (3%)	16 (2%)	12 (4%)
Distant recurrence	No	822 (85%)	552 (86%)	270 (83%)
	Yes	149 (15%)	92 (14%)	57 (17%)
Survival	Alive	697 (72%)	444 (69%)	253 (77%)
	Dead	274 (28%)	200 (31%)	74 (23%)

* Risk factors = Emergency surgery, fewer than 12 nodes investigated, vascular or perineural invasion, undifferentiated/high grade differentiation, and pT4.

Publisher's Note: MDPI stays neutral with regard to jurisdictional claims in published maps and institutional affiliations.

© 2020 by the authors. Licensee MDPI, Basel, Switzerland. This article is an open access article distributed under the terms and conditions of the Creative Commons Attribution (CC BY) license (http://creativecommons.org/licenses/by/4.0/).
